# Protective Effect of Pentoxifylline on the Development of Acute Gastric Mucosal Injury in a Model of LPS-Induced Sepsis

**DOI:** 10.3390/antiox13121481

**Published:** 2024-12-04

**Authors:** Sergio D. Paredes, Jorge Hernández-Cortés, Farzin Falahat, Lisa Rancan, Javier Arias-Díaz, Elena Vara

**Affiliations:** 1 Department of Physiology, School of Medicine, Complutense University of Madrid, Avda. Complutense, s/n, 28040 Madrid, Spain; 2 Department of Biochemistry and Molecular Biology, School of Medicine, Complutense University of Madrid, Avda. Complutense, s/n, 28040 Madrid, Spain; jorgehcortes@hotmail.com (J.H.-C.); lisaranc@ucm.es (L.R.); evaraami@ucm.es (E.V.); 3 Department of Surgery, School of Medicine, Complutense University of Madrid, Avda. Complutense, s/n, 28040 Madrid, Spain; ffalahat@ucm.es (F.F.); javierar@ucm.es (J.A.-D.); 4 Oral and Maxillofacial Surgery Service, Hospital Clínico San Carlos, 28040 Madrid, Spain

**Keywords:** pentoxifylline, phosphatidylcholine, myeloperoxidase, prostaglandin, thromboxane, leukotriene, lipid hydroperoxide, nitric oxide, gastric mucosa, LPS-induced sepsis

## Abstract

Alterations in the gastric mucosal barrier, one of whose fundamental components is phosphatidylcholine (PC), may play an important role in the pathophysiology of erosive gastritis secondary to sepsis. Pentoxifylline (PTX) has been shown to reduce tissue damage in various experimental models of sepsis. The aim of this study was to investigate the effect of PTX on gastric mucosa PC synthesis, leukocyte infiltration, arachidonic acid-related metabolites, inflammation, oxidative stress, NO, CO, and somatostatin in a rat model of LPS-induced sepsis. Rats were administered LPS (10 mg/kg b.w.) intraperitoneally. After 30 min (early treatment group) or 120 min (late treatment group) of LPS administration, they were randomly divided into two groups that were intraperitoneally administered saline (5 mL/kg; LPS + Saline group) or PTX (45 mg/kg; 5 mL/kg; LPS + PTX group). Control rats received only saline instead of LPS and/or PTX. Two hours after saline or PTX administration (total of 150 or 240 min of procedure), animals were anesthetized, and then gastric lavage, gastric mucosa and plasma samples were obtained and kept frozen until determination. LPS-induced sepsis changed the gastric mucosal barrier by reducing its phospholipid content, PGI2, and somatostatin levels, as well as increasing MPO, TXB2, LTB4, PLA2, and MDA. Alterations may be mediated, at least in part, by modifications in the production of NO, CO, and cGMP content. PTX had a beneficial effect on gastric lesions secondary to sepsis by restoring PC production.

## 1. Introduction

Shock can be defined as a hemodynamic alteration that will lead to cellular dysfunction due to the failure of its mechanisms for obtaining energy [1]. Shock states are perhaps one of the most complex problems of all surgical pathophysiology. Septic shock continues to have a high mortality rate and can be caused by bacterial infections, viruses, protozoa and fungi. Gram-negative bacterial infections are some of the most common among these microorganisms, with lipopolysaccharide (LPS) being widely acknowledged as a key external factor in triggering septic shock [2].

Septic shock contributes to a significant percentage of in-hospital deaths, including in intensive care units [3]. A number of patients with septic shock treated in intensive care units progress to refractory hypotension or progressive multiorgan failure. Sepsis, especially of abdominal origin, remains the most common predisposing factor for the latter syndrome [4]. One important manifestation of multiorgan failure is bleeding from acute ulcerations of the gastric mucosa (stress ulcers). This complication, even in isolation, continues to be associated with a high risk of mortality when it develops in septic or postoperative patients [5].

The role of neutrophils in the inflammatory process that occurs during sepsis is well known [6]. These cells adhere to the vascular endothelium, causing microthrombosis, and can also release a series of mediators, including phospholipase A2 (PLA2), activation of the arachidonic acid cascade, peroxidases and proteases and reactive oxygen species (ROS) [7]. These mediators, probably interrelated, are able to increase the inflammatory response and thus cause greater tissue injury, so a target for improving sepsis-induced morbidity and mortality would be the testing of therapies that act on ROS.

PTX, a methylxanthine phosphodiesterase inhibitor, reduces tissue damage in septic animals and is able to improve both hemodynamic manifestations and survival in experimental models of septic shock [8,9]. PTX can increase tissue blood flow in low-flow states through a direct vasodilator effect and by improving red cell deformability [10]. This property of maintaining tissue oxygenation has been proposed as the mechanism by which survival of hemorrhagic shock models treated with PTX is improved [11]. PTX has also been shown to be a protective agent against indomethacin-induced gastric mucosal injury in rats [12], exerting this effect by decreasing neutrophilic polymorphonuclear adhesion, unrelated to prostaglandin synthesis or possible inhibition of acid secretion [13]. Furthermore, it has been seen that in situations of ischemia, as in peripheral vascular disease, PTX is an agent that inhibits the synthesis of free radicals [14], and although this effect of PTX has not been shown to exist in mucosal injury secondary to sepsis, perhaps it could occur, given that the condition of hypoperfusion or ischemia exists.

The initial objective of this work was to investigate the synthesis of phosphatidylcholine (PC) and dipalmitoylphosphatidylcholine (DPPC) by the gastric mucosa in a rat model of LPS-induced sepsis, as well as the possible involvement of arachidonic acid metabolites (prostaglandin E2 [PGE2], prostaglandin I2 [PGI2], thromboxane B2 [TXB2], leukotriene B4 [LTB4]), myeloperoxidase (MPO) as an index of leukocyte infiltration, inflammation-related mediator PLA2, and malondialdehyde (MDA) in gastric mucosal damage. On the other hand, although nitric oxide (NO) exerts a mucosal protective role in physiological situations by favoring vasodilatation, the local production of significant amounts of NO could play an important role in the alterations that occur in the gastric mucosa during sepsis. NO seems to exert part of its effects by activation of guanylate cyclase and the consequent increase in cyclic guanosine monophosphate (cGMP) levels. Since carbon monoxide (CO) can act as an intracellular messenger, activating the soluble fraction of guanylate cyclase and sharing other properties of NO, our next objective was to investigate a possible role of local NO and CO production in gastric mucosal alterations. We also evaluated somatostatin levels. Finally, given that PTX has been able to reduce tissue damage in various models of sepsis, the possible protective effect that this molecule may exert on induced gastric mucosal alterations triggered by LPS was studied.

## 2. Materials and Methods

### 2.1. Animals

Male Wistar rats weighing between 250 and 300 g were housed in a facility where they were subjected to automatic control of light-dark cycles (12 h of light, 8–20 h and 12 h of darkness) and temperature (22 ± 20 °C), and fed with a standard diet supplied by Panlab (Barcelona, Spain) and water “ad libitum”. All animals received humane care, and the Ethical Norms for Animal Research, as dictated by the European Union, were strictly always followed (2010/63/UE).

### 2.2. Experimental Design

After rats were fasted overnight, they were administered Escherichia coli 055:BS LPS (Sigma Chemical Co., St Louis, MO, USA) at a dose of 10 mg/kg b.w. intraperitoneally. Although there is a consensus that no single ideal model of shock or sepsis exists [15], this sepsis model was selected due to its technical simplicity and reproducibility among the variety of available models [16,17,18]. In preliminary dose-response studies, we found that administering this specific E. coli LPS at a dose of 10 mg/kg resulted in approximately 30% survival of rats at 24 h. The survival rate represents a balance between inducing severe sepsis and maintaining some physiological compensatory mechanisms. Such a balance is crucial because it more closely mirrors severe human sepsis, where patients still have survival opportunities. By choosing this model and dosage, we aimed to create a condition that was sufficiently severe to test the potential protective effects of PTX yet not so lethal that it precluded the possibility of observing meaningful physiological responses.

After 30 min (10 rats/group; early treatment group) or 120 min (10 rats/group; late treatment group) of LPS administration, the rats were randomly divided into two subgroups, which were administered, also intraperitoneally, tritiated choline (20 µCi of [methyl-^3^H] choline), as a marker of PC synthesis, together with one of the following compounds: (1) saline (5 mL/kg; LPS + Saline groups) and (2) PTX (a single dose of 45 mg/kg; 5 mL/kg; LPS + PTX groups).

Thus, experimental groups were organized as follows:

(1) Group 1: animals treated with LPS for 30 min followed by treatment of saline for 120 min (early treatment).
(2) Group 2: animals treated with LPS for 30 min followed by treatment of PTX for 120 min (early treatment).
(3) Group 3: animals treated with LPS for 120 min followed by treatment of saline for 120 min (late treatment).
(4) Group 4: animals treated with LPS for 120 min followed by treatment of PTX for 120 min (late treatment).

Monitoring of the results was performed up to 150 min (30 min LPS + 120 min saline or PTX treatment; groups 1 and 2, respectively) or 240 min (120 min LPS + 120 min saline or PTX treatment; groups 3 and 4, respectively).

The animals assigned to the control group were handled in exactly the same way as those allocated to the experimental groups, administering saline instead of LPS or PTX.

The selection of a 45 mg/kg dose of PTX for administration to Wistar rats in the present study was based on previous studies demonstrating the efficacy and safety of a similar dose range in rat models [19,20].

### 2.3. Sample Collection

Two hours after administration of tritiated choline plus saline or PTX treatments (i.e., 150 or 240 min after LPS administration), the rats were anesthetized with ketamine (10 mg/kg; 2 mL/kg) injected intraperitoneally, and blood, gastric lavage and gastric mucosa samples were obtained.

To obtain the gastric lavage samples, after performing the laparotomy, the stomach was dissected, and its exit was ligated at the pylorus level. After sectioning the esophago-gastric junction, a 14-gauge Teflon catheter connected to a syringe was introduced through it, and the gastric cavity was washed with 2 mL of 0.9% NaCl solution (4 °C). The liquid, which was recovered with the same syringe, was frozen at −80 °C until the determination of its NO_2_^−^ + NO_3_^−^, CO and protein content.

For the gastric mucosa determinations, after complete removal of the stomach, this organ was opened longitudinally through its lesser curvature and the gastric mucosa was scraped with a curette, divided into several tubes and stored frozen (−80 °C) until the time of measurement of the different parameters investigated.

### 2.4. Phosphatidylcholine (PC) and Dipalmitoylphosphatidylcholine (DPPC)

For PC extraction, a mucosal sample was homogenized in chloroform/methanol (2:1) to a final dilution of 1/17. The mixture was then equilibrated at room temperature for 1 h and filtered into a ground glass tube with a ground glass stopper. The crude extract was mixed with 0.2 times the volume of Folch’s solution, and the two phases of the mixture were separated by centrifugation. After aspirating the upper phase, three washes were performed with UPS (50% of the original volume), centrifuging after each wash and discarding the upper phase. Finally, the lower phase and the remnant of the upper phase were converted to one phase by the addition of methanol, and the resulting solution was evaporated under vacuum. The dried extract was redissolved again with chloroform/methanol and transferred to a scintillation vial to determine its radioactivity in a liquid scintillation counter. The results were expressed as cpm/mg protein.

To separate the disaturated form of PC (DPPC), the complete PC fraction was reacted with osmium tetroxide in carbon tetrachloride, and the disaturated PC species were separated from the unsaturated ones by thin layer chromatography on boric acid-impregnated silica gel plates using a mixture of chloroform/methanol/ammonium hydrochloride/H_2_O (75:25:1:2 *v*/*v*). As a control, different amounts of a standard DPPC solution were applied directly to the plate. The procedure was repeated to calculate the recovery of disaturated species from the sample with radioactive saturated PC samples. The recovery was 79.5 ± 6.2% (n = 6).

### 2.5. Myeloperoxidase (MPO)

MPO was determined by the modified Bradley method [21]. For this purpose, a sample of gastric mucosa was homogenized in phosphate buffer (pH 6.0) and centrifuged at 20,000× *g* for 15 min. The supernatant was discarded, and the precipitate was resuspended in phosphate buffer with hexadecyltrimethylammonium bromide to remove possible pseudoperoxidase activity and solubilize membrane-bound MPO. This suspension was sonicated for 20 s, frozen and thawed three times. An aliquot of the final supernatant was mixed with phosphate buffer containing O-dianisidine dihydrochloride and hydrogen peroxide, and its absorbance was determined at 460 nm.

### 2.6. Arachidonic Acid Metabolites: Prostaglandin E2 (PGE2), Prostaglandin I2 (PGI2), Thromboxane B2 (TXB2), and Leukotriene B4 (LTB4)

They were measured by specific RIA kits (Amersham, England, UK). After specific extraction, all samples were processed immediately after collection. Given its instability, PGI2 was determined as the concentration of its stable metabolite 6-keto-PGFlα. PGE2 was converted into its methyl oxymate derivative.

### 2.7. Proteins

Protein determination was performed by a colorimetric method described by Bradford [22]. The basis of this method is the binding of Coomassie Brilliant Blue to proteins. This binding produces a shift of the absorption maximum of the dye from 465 to 595 nm. The absorbance of the samples at the latter wavelength is monitored against a known standard curve. The protein-dye complex has a high extinction coefficient, which provides a high sensitivity in protein measurement.

### 2.8. Phospholipase A2 (PLA2)

PLA2 activity was measured as ^3^H-arachidonate release using a suspension of Escherichia coli labeled with ^3^H-arachidonate as substrate [23]. After homogenization of the tissue in phosphate buffer saline (PBS) containing 0.1 mmol/L phenylmethylsulfonylfluoride, aliquots (200 µL) of the samples were incubated at 37 °C for 1 h in buffer containing 0.1% Triton x-100, 40 mmol Trizma base, 0.65 mmol/L deoxycholic acid, 2 mmol/L CaCl_2_, and 0.25 µCi of the labeled E. coli suspension, pH 7.5. The reaction was stopped by the addition of 2 mL of 2-propanol/n-heptane/1 mol/L H_2_SO_4_ (40:10:1). Then, 2 mL of n-heptane and 3 mL of distilled water were added for the formation of two phases, and the radioactivity of the upper phase was determined in a liquid scintillation counter. PLA2 activity was expressed as IU/mg protein. One unit is equivalent to 1% of the total counts.

### 2.9. Lipid Peroxidation

It was determined as the production of malondialdehyde (MDA) by thiobarbituric acid (TBA) reaction. For this purpose, the sample was homogenized in PBS. The resulting suspension was centrifuged (3000× *g*, 10 min) and an aliquot of the supernatant was taken which, after being deproteinized, was left to react for 12 h with a solution containing hydrochloric acid (0.25 mol/L), trichloroacetic acid (15%), thiobarbituric acid (3 mmol/L) and 2-tetrabutyryl-4-methylphenol (0.1%). After 12 h of reaction, its absorbance was measured at 533 nm, using 1-1-3-3-tetraethoxypropane as standard.

### 2.10. NO, Nitrate Synthase (NOS) Activity, Nitrosothiols, cGMP, and CO

NO was determined as nitrite (NO_2_^−^) content. For this purpose, the samples were deproteinized by the addition of sulfosalicylic acid and incubated for 30 min at 40 °C. After centrifugation for 20 min (12,000× *g*), the supernatant was separated and incubated in the presence of nitrate reductase for the reduction of NO_3_^−^ to NO_2_^−^. Griess reagent was then added to all samples, and their absorbance was measured at 546 nm, using NaNO_2_ solution as a standard.

NOS activity was measured as the conversion of ^14^C-Arginine to citrulline. Briefly, tissue samples were homogenized in a buffer containing 10^−2^ mol/L Hepes, 0.32 mol/L sucrose, 10^−4^ mol/L EDTA, 10^−3^ mol/L dithiothreitol, 10 µg/mL leupeptin, 2 µg/mL aprotinin, and 1 mg/mL phenylmethanesulfonylfluoride. The samples were then centrifuged at 100,000× *g*, at 40 °C, for 20 min, and an aliquot of the supernatant was incubated in the presence of the radioactive precursor for 30 min at 37 °C, and after purification by ion exchange on a Dowes AG 8 resin column, radioactivity was measured in a liquid scintillation counter.

The Saville method was used for the determination of nitrosothiols (NO + NOSH) [24]. This method is based on the binding of the Hg^2+^ cation to S, forming a complex susceptible to nucleophilic attack by H_2_O molecules.

cGMP was measured using specific RIA kits (NEN, Boston, MA, USA). Briefly, after the addition of isomethyl buthyl xanthine (phosphodiesterase inhibitor), the tissue was manually homogenized in a glass homogenizer and then sonicated with an ultrasound disruptor. Proteins were precipitated, and the cyclic nucleotide was extracted with ethanol (80% *v*/*v*). After centrifugation, the supernatant was separated into two aliquots, which were transferred to RIA tubes and evaporated at 37 °C. Residues were reconstituted with an RIA buffer and determined by RIA following the specific instructions for each kit. The recovery of [^3^H] GMPc was 97.6 ± 2.1% (n = 6).

To quantify the amount of CO formed, hemoglobin (Hb), which binds CO, was added, and the amount of carboxyhemoglobin (COHb) was estimated. For this, Hb was added to the sample, and a wait time of 1 min was allowed to ensure maximum CO binding. The samples were then diluted with a phosphate buffer containing sodium dithionite and allowed to react for 10 min at room temperature. Their absorbance was read at 421 and 432 nm, using a sample containing only buffer as a blank.

### 2.11. Somatostatin

The buffer used for somatostatin RIA had the following composition: Na_2_HPO_4_ 0.04 mol/L, NaCl 0.14 mol/L and EDTA 0.025 mol/L, bovine albumin 0.25%, pH = 7.4. A total of 100 µL of diluted somatostatin-specific antibody, 500 µL of Na^125^I-labeled somatostatin, 100 µL of sample or else somatostatin standard solutions were pipetted into 4-mL tubes and incubated at 4 °C for 48 h. Separation of free somatostatin from that bound to the antibody was performed by adding 1 mL of a carbon-dextran suspension (0.25% carbon and 0.025% dextran) in phosphate buffer, containing 0.9% NaCl to all tubes except those containing only total activity. Finally, the radioactivity of the precipitate was measured in a gamma particle counter. The sensitivity of the radioimmunoassay was 0.03202 pg/tube, and the zone of minimum error corresponded to the concentration range between 10 and 80 pg/tube. A specific in-house antibody [25] at a final dilution of 1/56,000 was used in all determinations.

### 2.12. Statistical Analysis

Nonparametric statistical tests were used. The results were expressed as mean ± SEM. For comparisons between groups, the Kruskal-Wallis test for analysis of variance (ANOVA) by ranks was used, followed, if significant, by the Mann-Whitney test for independent samples to identify the origin of the differences. Confidence values equal to or greater than 95% (*p* < 0.05) and 99% (*p* < 0.01), respectively, were considered significant and highly significant.

## 3. Results

### 3.1. Effect of LPS and PTX Treatment on PC Synthesis

**Figure 1:**
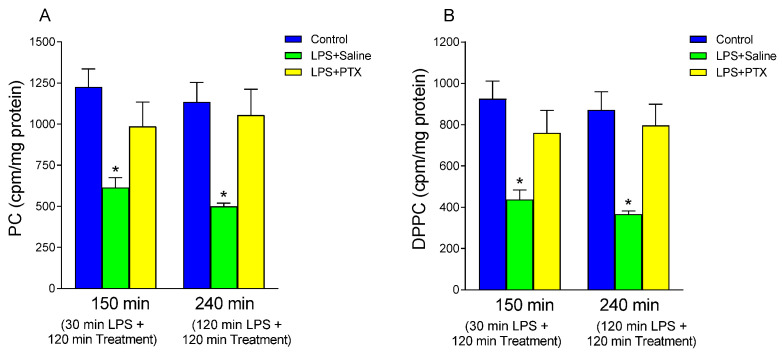
The figure shows the content of PC (cpm/mg protein) (**A**) and DPPC (cpm/mg protein) (**B**) in gastric mucosa of male Wistar rats after 30 min of LPS administration + 120 min of treatment (early treatment): saline (LPS + Saline group; green columns) or PTX (LPS + PTX group; yellow columns); total of 150 min of procedure; or 120 min of LPS administration + 120 min of treatment (late treatment): saline (LPS + Saline group; green columns) or PTX (LPS + PTX group; yellow columns); total of 240 min of procedure. Control animals (blue columns) received only saline instead of LPS and/or PTX. (*) *p* < 0.01 vs. the rest of the groups.

To study the effect of bacterial LPS on the synthesis of PC by gastric mucosal cells, the incorporation of labeled choline ([methyl-^3^H] choline) into PC extracted from gastric tissue was determined. As shown in Figure 1A, LPS administration induced a significant decrease in the incorporation of labeled choline into PC, with respect to the control group, both at 150 min (613.300 ± 60.663 vs. 1225.50 ± 110.541 cpm/mg protein, *p* < 0.01; n = 10) and 240 min (498.778 ± 19.976 vs. 1134.33 ± 119.778 cpm/mg protein, *p* < 0.01; n = 9) after treatment. When PTX was administered 30 min after LPS, these effects were blocked (Figure 1A; a total of 150 min of procedure). Thus, PTX induced increased incorporation of labeled choline into PC (986.00 ± 148.507 vs. 613.300 ± 60.663 cpm/mg protein, *p* < 0.01; n = 10). Treatment with PTX at 120 min after LPS administration also blocked the effect of LPS on the incorporation of labeled choline into PC from gastric mucosa (1054.00 ± 158.032 vs. 498.77 ± 19.976 cpm/mg protein, *p* < 0.01; n = 9) (Figure 1A; total of 240 min of procedure).

Since the PC fraction of the gastric mucosal barrier is rich in its saturated form DPPC, we decided to study next whether changes in PC labeling reflected changes in the labeling of the saturated form. As seen in Figure 1B, LPS decreased DPPC synthesis by mucosal cells vs. the control group at 150 min (437.00 ± 46.358 vs. 925.50 ± 85.531 cpm/mg protein, *p* < 0.01; n = 10) and at 240 min (366.00 ± 16.151 vs. 871.667 ± 88.362 cpm/mg protein, *p* < 0.01; n = 9). This effect was totally blocked by PTX in both early and late treatment groups (760.00 ± 109.319 vs. 437.00 ± 46.358 cpm/mg protein, *p* < 0.01; n = 10; and 796.444 ± 102.892 vs. 366.00 ± 16.151 cpm/mg protein, *p* < 0.01; n = 9, after 30 or 120 min of LPS administration) without finding significant differences with respect to the control group.

### 3.2. Effect of LPS and PTX Treatment on MPO

**Figure 2:**
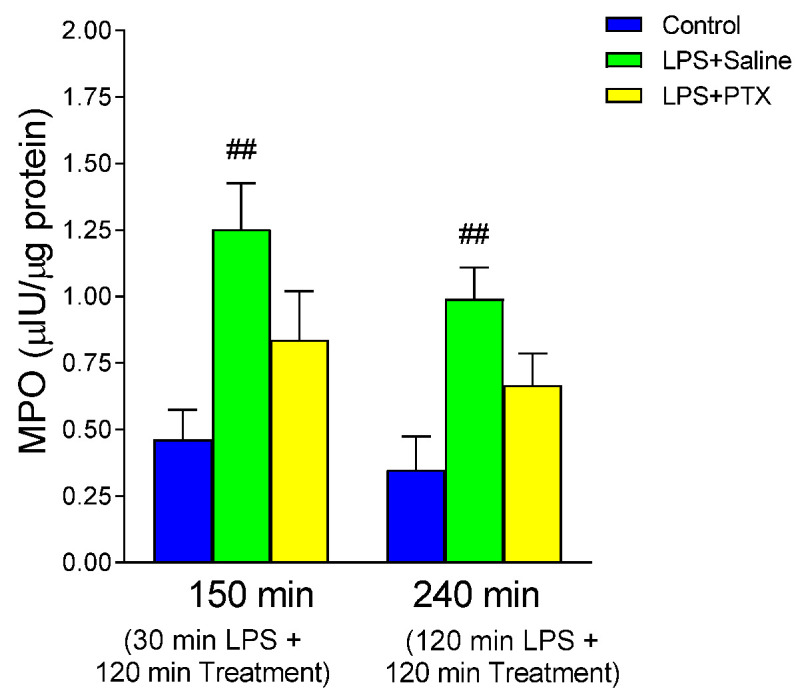
The figure shows MPO activity (µIU/µg protein) in gastric mucosa of male Wistar rats after 30 min of LPS administration + 120 min of treatment (early treatment): saline (LPS + Saline group; green columns) or PTX (LPS + PTX group; yellow columns); total of 150 min of procedure; or 120 min of LPS administration + 120 min of treatment (late treatment): saline (LPS + Saline group; green columns) or PTX (LPS + PTX group; yellow columns); total of 240 min of procedure. Control animals (blue columns) received only saline instead of LPS and/or PTX. (##) *p* < 0.01 vs. the control groups.

To determine the degree of leukocyte infiltration, MPO content in gastric tissue was measured (Figure 2). Intraperitoneal injection of LPS caused a significant increase in MPO, both at 150 min (1.253 ± 0.174 vs. 0.462 ± 0.113 µIU/µg protein, *p* < 0.01; n = 10) and at 240 min (0.991 ± 0.119 vs. 0.3488 ± 0.1246 µIU/µg protein, *p* < 0.01; n = 9). The administration of PTX did not significantly modify the MPO activity with respect to the LPS group, even though the values obtained were lower.

### 3.3. Effect of LPS and PTX Treatment on Arachidonic Acid Metabolites

It is known that the gastric mucosa releases a series of mediators capable of protecting or aggravating tissue aggression; among them are the arachidonic acid metabolites that are considered to be involved in the pathogenesis of stress ulcers. PGE2 has been attributed a cytoprotective role in the gastric mucosa; on the other hand, PGI2 and TXB2, also derived from the cyclooxygenase pathway, have antagonistic roles. TXB2 is the stable metabolite of thromboxane A2 (TXA2), has a vasoconstrictor action and stimulates platelet aggregation. In contrast, PGI2 exerts a vasodilator action and inhibits platelet aggregation.

**Figure 3:**
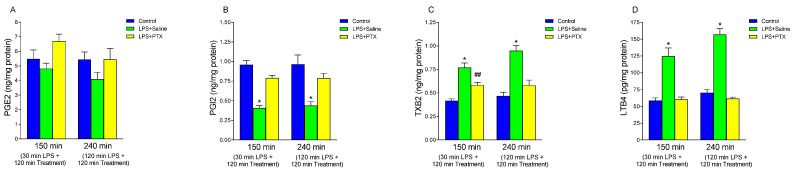
The figure shows the content of PGE2 (ng/mg protein) (**A**), PGI2 (ng/mg protein) (**B**), TXB2 (ng/mg protein) (**C**), and LTB4 (pg/mg protein) (**D**) in gastric mucosa of male Wistar rats after 30 min of LPS administration + 120 min of treatment (early treatment): saline (LPS + Saline group; green columns) or PTX (LPS + PTX group; yellow columns); total of 150 min of procedure; or 120 min of LPS administration + 120 min of treatment (late treatment): saline (LPS + Saline group; green columns) or PTX (LPS + PTX group; yellow columns); total of 240 min of procedure. Control animals (blue columns) received only saline instead of LPS and/or PTX. (*) *p* < 0.01 vs. rest of the groups; (##) *p* < 0.01 vs. the control groups.

Neither intraperitoneal injection of LPS nor administration of PTX significantly changed the concentration of PGE2 in the gastric mucosa (Figure 3A). However, intraperitoneal injection of LPS induced a significant decrease in gastric tissue production of PGI2 (Figure 3B), both at 150 min (0.408 ± 0.030 vs. 0.957 ± 0.0548 ng/mg protein, *p* < 0.01; n = 10) and at 240 min (0.4388 ± 0.049 vs. 0.963 ± 0.1195 ng/mg protein, *p* < 0.01; n = 9). This effect was blocked by PTX, both at 150 min (0.790 ± 0.033 vs. 0.408 ± 0.030 ng/mg protein, *p* < 0.01; n = 10) and 240 min (0.7866 ± 0.063 vs. 0.4388 ± 0.049 ng/mg protein, *p* < 0.01; n = 9) (Figure 3B).

As seen in Figure 3C, LPS induced a significant increase in tissue production of TXB2 relative to the control group at 150 (0.769 ± 0.0495 vs. 0.416 ± 0.0199 ng/mg protein, *p* < 0.01; n = 10) and 240 min (0.9466 ± 0.056 vs. 0.4677 ± 0.039 ng/mg protein, *p* < 0.01; n = 9). PTX treatment significantly reduced the effects of LPS (0.580 ± 0.0318 vs. 0.769 ± 0.0495 ng/mg protein, *p* < 0.01; n = 10, and 0.580 ± 0.0559 vs. 0.9466 ± 0.056 ng/mg protein, *p* < 0.01; n = 9, with early and late treatment respectively), although the blockade observed 30 min after LPS injection (total of 150 of procedure) was partial, finding statistically higher TXB2 values than those of the control group (0.580 ± 0.0318 vs. 0.416 ± 0.0199 ng/mg protein, *p* < 0.01; n = 10).

Finally, arachidonic acid can be metabolized by alternative pathways to that of cyclooxygenase, including the lipooxygenase pathway. Given the attributed importance of leukotrienes in the pathogenesis of stress ulcers, consisting in favoring damage on the gastric mucosa (perhaps by stimulating ROS production and facilitating neutrophil chemotactic and adhesion activity), we studied whether LPS modified LTB4 levels in the gastric mucosa (Figure 3D). Intraperitoneal injection of LPS induced a significant increase of LTB4 with respect to the control groups, both at 150 min (124.609 ± 12.404 vs. 58.406 ± 4.179 pg/mg protein, *p* < 0.01; n = 10) and at 240 min (156.854 ± 8.909 vs. 70.302 ± 5.086 pg/mg protein, *p* < 0.01; n = 9). Treatment with PTX after 30 min of LPS administration [total of 150 min of procedure] (60.301 ± 3.546 vs. 124.609 ± 12.404 pg/mg protein, *p* < 0.01; n = 10), or after 120 min of LPS administration [total of 240 min of procedure] (61.454 ± 2.372 vs. 156.854 ± 8.909 pg/mg protein, *p* < 0.01; n = 9) completely reversed the effect observed with LPS.

### 3.4. Effect of LPS and PTX Treatment on PLA2

**Figure 4:**
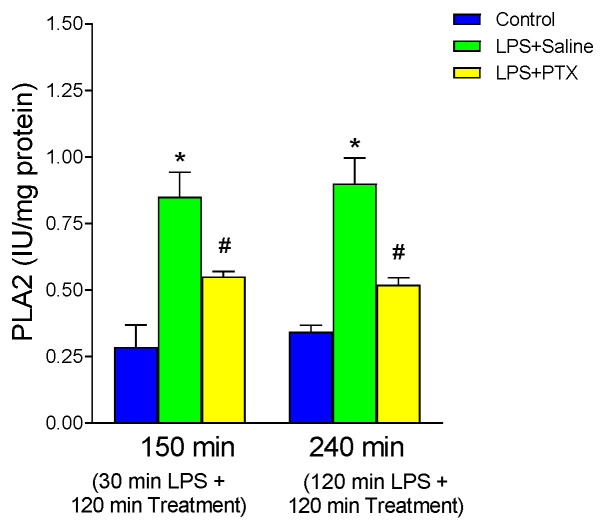
The figure shows PLA2 activity (IU/mg protein) in gastric mucosa of male Wistar rats after 30 min of LPS administration + 120 min of treatment (early treatment): saline (LPS + Saline group; green columns) or PTX (LPS + PTX group; yellow columns); total of 150 min of procedure; or 120 min of LPS administration + 120 min of treatment (late treatment): saline (LPS + Saline group; green columns) or PTX (LPS + PTX group; yellow columns); total of 240 min of procedure. Control animals (blue columns) received only saline instead of LPS and/or PTX. (*) *p* < 0.01 vs. rest of the groups; (#) *p* < 0.05 vs. the control groups.

PLA2 is known to play an important role in mediating inflammatory phenomena during sepsis. In addition, bacterial LPS activates PLA2 of cell membranes, and thus phospholipid hydrolysis occurs, which is a limiting step in the release of arachidonic acid. Therefore, we determined the activity of this enzyme in gastric mucosa (Figure 4). After LPS administration, a significant increase in PLA2 activity was observed with respect to the control group at both 150 and 240 min (0.850 ± 0.0927 vs. 0.285 ± 0.083 IU/mg protein, *p* < 0.01; n = 5, and 0.900 ± 0.0958 vs. 0.3425 ± 0.0245 IU/mg protein, *p* < 0.01; n = 5). PTX administration induced a significant decrease in the activity of this enzyme in gastric mucosa, both 30 min after LPS [total of 150 min of procedure] (0.55 ± 0.020 vs. 0.850 ± 0.0927 IU/mg protein, *p* < 0.01; n = 5) and 120 min after LPS [total of 240 min of procedure] (0.520 ± 0.0258 vs. 0.900 ± 0.0958 IU/mg protein, *p* < 0.01; n = 5) although the blockade with this substance was partial, obtaining a significantly higher enzyme activity than in the control group (0.550 ± 0.020 vs. 0.285 ± 0.083 IU/mg protein, *p* < 0.05; n = 5, and 0.520 ± 0.0258 vs. 0.3425 ± 0.0245 IU/mg protein, *p* < 0.05; n = 5, early and late treatment respectively).

### 3.5. Effect of LPS and PTX Treatment on MDA

**Figure 5:**
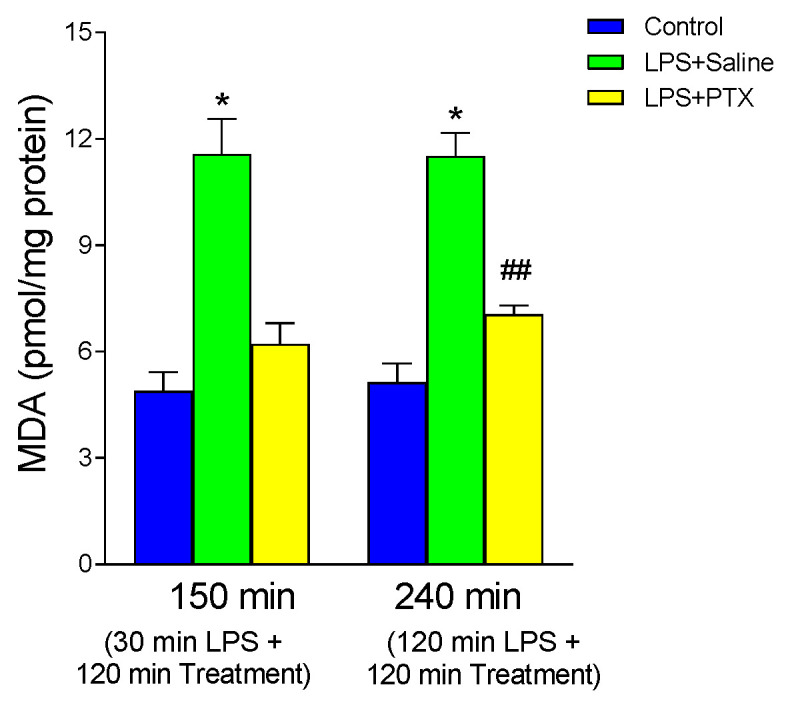
The figure shows the content of MDA (pmol/mg protein) in gastric mucosa of male Wistar rats after 30 min of LPS administration + 120 min of treatment (early treatment): saline (LPS + Saline group; green columns) or PTX (LPS + PTX group; yellow columns); total of 150 min of procedure; or 120 min of LPS administration + 120 min of treatment (late treatment): saline (LPS + Saline group; green columns) or PTX (LPS + PTX group; yellow columns); total of 240 min of procedure. Control animals (blue columns) received only saline instead of LPS and/or PTX. (*) *p* < 0.01 vs. rest of the groups; (##) *p* < 0.01 vs. the control groups.

Figure 5 shows the production of MDA in the gastric mucosa as an index of lipid peroxidation, an action attributed to oxygen-free radicals produced by polymorphonuclear leukocytes. LPS induced a significant increase in MDA production with respect to the control group, both at 150 min (11.565 ± 0.997 vs. 4.884 ± 0.533 pmol/mg protein, *p* < 0.01; n = 10) and at 240 min (11.5089 ± 0.653 vs. 5.132 ± 0.534 pmol/mg protein, *p* < 0.01; n = 9). Treatment with PTX, after 30 min of LPS administration (total of 150 min of procedure), completely blocked the effect of LPS (6.216 ± 0.579 vs. 11.565 ± 0.997 pmol/mg protein, *p* < 0.01). However, late PTX administration reversed the effect of LPS only partially (7.0477 ± 0.2527 vs. 11.5089 ± 0.653 pmol/mg protein, *p* < 0.01), finding significantly higher values than controls (7.0477 ± 0.2527 vs. 5.132 ± 0.534 pmol/mg protein, *p* < 0.01, a total of 240 min of procedure).

### 3.6. Effect of LPS and PTX Treatment on NO, NOS Activity, Nitrosothiols, cGMP, and CO

**Figure 6:**
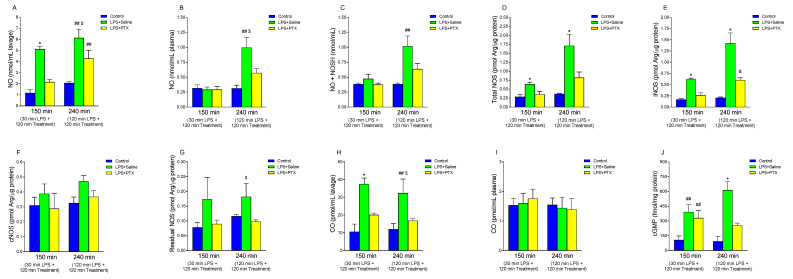
The figure shows NO concentration in gastric lavage (nmol/mL lavage) (**A**), and plasma (nmol/mL plasma) (**B**), and CO concentration in gastric lavage (pmol/mL lavage) (**H**), and plasma (pmol/mL plasma) (**I**), as well as NO + NOSH content (nmol/mL) (**C**), total NOS activity (pmol Arg/µg protein) (**D**), iNOS activity (pmol Arg/µg protein) (**E**), cNOS activity (pmol Arg/µg protein) (**F**), residual NOS (pmol Arg/µg protein) (**G**), and cGMP content (fmol/mg protein) (**J**) in gastric mucosa of male Wistar rats after 30 min of LPS administration + 120 min of treatment (early treatment): saline (LPS + Saline group; green columns) or PTX (LPS + PTX group; yellow columns); total of 150 min of procedure; or 120 min of LPS administration + 120 min of treatment (late treatment): saline (LPS + Saline group; green columns) or PTX (LPS + PTX group; yellow columns); total of 240 min of procedure. Control animals (blue columns) received only saline instead of LPS and/or PTX. (*) *p* < 0.01 vs. rest of the groups; (#) *p* < 0.05 or (##) *p* < 0.01 vs. the control groups; ($) *p* < 0.05 vs. LPS + PTX.

NO concentration was determined in both gastric lavage fluid and plasma (Figure 6A and Figure 6B, respectively). In gastric lavage fluid, LPS significantly increased NO levels at both 150 min (5.112 ± 0.2635 vs. 1.168 ± 0.301 nmol/mL lavage, *p* < 0.01; n = 5) and 240 min (6.13 ± 0.794 vs. 2.064 ± 0.139 nmol/mL lavage, *p* < 0.01; n = 5) relative to controls (Figure 6A). PTX treatment 30 min after LPS administration (total of 150 min of procedure) totally blocked this effect (2.152 ± 0.2479 vs. 5.112 ± 0.2635 nmol/mL lavage, *p* < 0.01), but PTX injection 120 min after LPS (total of 240 min of procedure) only partially blocked its effect (4.278 ± 0.7419 vs. 6.134 ± 0.794 nmol/mL lavage, *p* < 0.05; n = 5) (Figure 6A). On the other hand, LPS administration did not induce significant differences in plasma NO levels at 150 min, and neither did PTX treatment cause any alterations in these levels (Figure 6B). However, at 240 min, a significant increase in plasma NO concentration was obtained after intraperitoneal injection of LPS with respect to the control group (0.994 ± 0.173 vs. 0.313 ± 0.053 nmol/mL plasma, *p* < 0.01). Treatment with PTX partially reversed this effect (0.570 ± 0.0749 vs. 0.994 ± 0.173 nmol/mL plasma, *p* < 0.05; n = 5).

Since it seems that part of the biological actions of NO could be mediated not directly through free NO but through carrier molecules, nitrosothiols, and since no differences were seen in plasma levels of NO_3_^−^ or NO_2_^−^, we determined the concentration of nitrosothiols to rule out that there was an increased production of NO that went unnoticed due to being in this biological form. As we have described for NO, LPS administration induced a significant increase in NO + NOSH levels at 240 min vs. the control group (1.017 ± 0.173 vs. 0.3836 ± 0.0176 nmol/mL, *p* < 0.01; n = 5) with no significant differences found at 150 min (Figure 6C). PTX administration did not induce significant differences in nitrosothiol production by gastric mucosa, although a downward trend was observed with late treatment.

The values of NOS activity are shown in Figure 6D. When LPS was administered, there was a significant increase in this activity, both after 30 min (total of 150 min of procedure) and after 120 min (total of 240 min of procedure), with respect to controls (0.636 ± 0.0578 vs. 0.286 ± 0.055 pmol Arg/µg protein, *p* < 0.01; n = 5, and 1.708 ± 0.321 vs. 0.366 ± 0.010 pmol Arg/µg protein, *p* < 0.01; n = 5). PTX administration induced a decrease in the activity of this enzyme, observing values similar to controls, both at 150 (0.356 ± 0.082 vs. 0.636 ± 0.0578 pmol Arg/µg protein, *p* < 0.01; n = 5) and 240 min (0.820 ± 0.156 vs. 1.708 ± 0.321 pmol Arg/µg protein, *p* < 0.01; n = 5). To determine the degree of involvement of each isoform, the inducible (Ca^2+^-dependent, iNOS) (Figure 6E) and constitutive (Ca^2+^-independent, cNOS) (Figure 6F) NOS activities were studied separately. We observed that after the injection of LPS, there was a significant increase in the inducible isoform, both at 150 and 240 min, compared to the control groups (0.618 ± 0.02478 vs. 0.166 ± 0.01887 pmol Arg/µg protein, *p* < 0.01; n = 5, and 1.418 ± 0.234 vs. 0.206 ± 0.0186 pmol Arg/µg protein, *p* < 0.01; n = 5, after 150 or 240 min respectively) (Figure 6E). When PTX was administered, a decrease in iNOS activity was observed with both treatment modalities, early (0.258 ± 0.0586 vs. 0.618 ± 0.02478 pmol Arg/µg protein, *p* < 0.01; n = 5) and late (0.600 ± 0.053 vs. 1.418 ± 0.234 pmol Arg/µg protein, *p* < 0.01; n = 5), although with late treatment the blockage was only partial, with significant differences compared to the control group (0.600 ± 0.053 vs. 0.206 ± 0.0186 pmol Arg/µg protein, *p* < 0.05; n = 5) (Figure 6E). In the case of cNOS (Figure 6F), although it showed a tendency to be higher after the administration of LPS and to normalize with the administration of PTX, no significant differences were observed. When we determined the residual nitrate synthase activity, the effects were similar (Figure 6G).

Plasma CO levels are depicted in Figure 6I. Like what was generally observed in the case of NO, no significant differences were found in any of the experimental situations studied. However, LPS induced a significant increase in CO levels in the gastric lavage fluid, compared to the control group, after 150 min (37.520 ± 3.3706 vs. 10.592 ± 4.329 pmol/mL lavage, *p* < 0.01; n = 5) or 240 min (32.448 ± 7.8859 vs. 12.048 ± 3.1468 pmol/mL lavage, *p* < 0.01; n = 5) of its administration (Figure 6H). Treatment with PTX 30 min after LPS (total of 150 min of procedure) induced a decrease in CO concentration in gastric lavage (20.096 ± 1.0425 vs. 37.520 ± 3.3706 pmol/mL lavage, *p* < 0.01; n = 5), blocking the effect of LPS. When PTX was administered 2 h after LPS (total of 240 min of procedure), the CO concentration in gastric lavage also significantly decreased (16.800 ± 1.17085 vs. 32.448 ± 7.8859 pmol/mL lavage, *p* < 0.05; n = 5) (Figure 6H).

The administration of LPS significantly increased the cGMP concentration at both 150 and 240 min (392.02 ± 74.815 vs. 107.18 ± 40.617 fmol/mg protein, *p* < 0.01; n = 5, and 613.76 ± 84.76 vs. 92.76 ± 49.76 fmol/mg protein, *p* < 0.01; n = 5, at 150 and 240 min respectively) (Figure 6J). After treatment with PTX, this effect was completely reversed after 240 min (253.74 ± 24.66 vs. 613.76 ± 84.76 fmol/mg protein, *p* < 0.01). However, in the early treatment group (total of 150 min of procedure), it was observed that, although after intraperitoneal injection of PTX, the concentration of cGMP seemed to be lower than that of the LPS group, there were no significant differences.

### 3.7. Effect of LPS and PTX Treatment on Somatostatin

**Figure 7:**
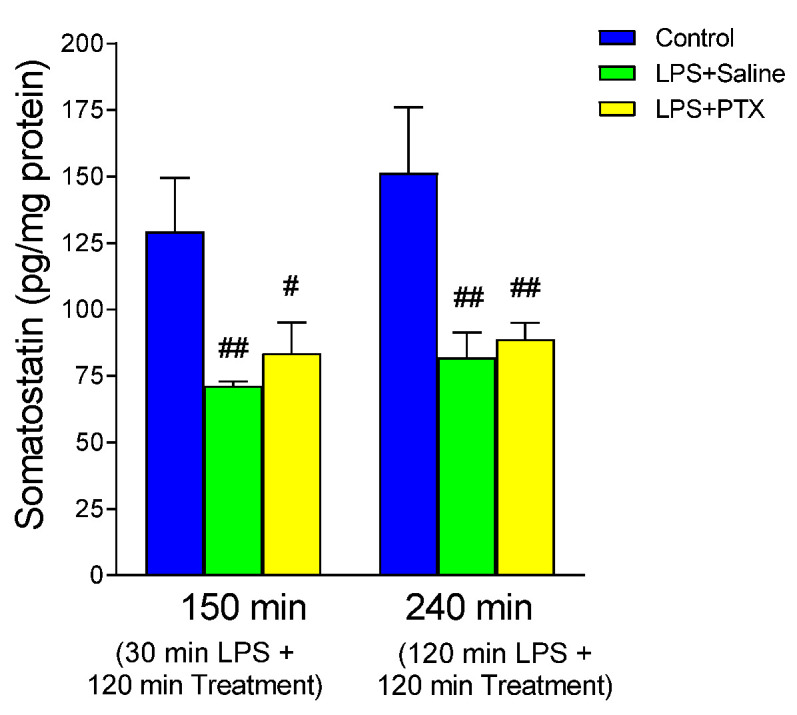
The figure shows the content of somatostatin (pg/mg protein) in gastric mucosa of male Wistar rats after 30 min of LPS administration + 120 min of treatment (early treatment): saline (LPS + Saline group; green columns) or PTX (LPS + PTX group; yellow columns); total of 150 min of procedure; or 120 min of LPS administration + 120 min of treatment (late treatment): saline (LPS + Saline group; green columns) or PTX (LPS + PTX group; yellow columns); total of 240 min of procedure. Control animals (blue columns) received only saline instead of LPS and/or PTX. (#) *p* < 0.05 or (##) *p* < 0.01 vs. the control groups.

LPS significantly decreased levels of somatostatin in the gastric mucosa (Figure 7). Treatment with PTX, 30 or 120 min after administration of LPS (total of 150 or 240 min of procedure, respectively), did not significantly modify the effect produced by LPS, and although slightly higher levels were detected than those obtained after LPS, levels remained statistically different from the control groups (83.540 ± 11.5906 vs. 129.200 ± 20.276 pg/mg protein, *p* < 0.05; n = 5, and 88.800 ± 6.2047 vs. 151.280 ± 24.768 pg/mg protein, *p* < 0.01; n = 5, early or late treatment with PTX respectively).

## 4. Discussion

Bleeding from stress ulcers, though less common recently, remains significant in septic patients and is viewed as part of intestinal failure in multiple organ failure syndrome [26]. The integrity of the gastric mucosal barrier is crucial for protection, with PC, the main phospholipid in gastric mucus, helping to maintain its hydrophobicity and shield it from harmful agents [27]. Likewise, PC has been shown to help preserve mucosal integrity during inflammatory conditions, such as those induced by LPS in animal models, further supporting its protective role in the gastrointestinal system [28]. Our results showed a decrease in the PC concentration of gastric mucus in animals subjected to the action of LPS compared to controls, as well as DPPC, which was expected since it is a form of PC that is mainly found in the lung and in the stomach, representing approximately 30% of gastric PC, and this was reverted by PTX treatment. PTX has shown anti-inflammatory effects that can indirectly contribute to the preservation of PC in tissues, although there are limited direct studies on this specific interaction. PTX is known for its role in inhibiting the production of inflammatory cytokines like TNF-α, which are associated with cellular injury, including in the gastrointestinal and renal systems [29]. Its ability to reduce inflammation and protect cell structures could support the maintenance of phospholipids such as PC by reducing the damage to cell membranes during inflammatory states. In the context of kidney disease, PTX has been shown to prevent the decline of important cellular proteins, like Klotho, which may provide a clue that its anti-inflammatory effects could extend to the protection of phospholipid layers like those in the gastric mucosa [30].

Sepsis from Gram-negative bacteria is linked to increased serum PLA2 activity, making it a mediator in septic shock. PLA2, found in cells like neutrophils and intestinal mucosa, is activated by bacterial endotoxin, leading to phospholipid hydrolysis. This releases arachidonic acid and generates products like leukotrienes and prostaglandins. PLA2 also degrades surfactant, leading to lysophosphatidylcholine accumulation in respiratory distress [31]. The same could occur in the gastric mucosa, whereby PLA2 would accelerate the degradation of PC, thus reducing its concentration at the gastric mucosal barrier, as this can occur both in physiological conditions and in situations of sepsis [32].

MPO activity indicates leukocyte infiltration. Neutrophils are crucial in inflammation and tissue damage during sepsis, including in the gastric mucosa. In sepsis, neutrophils aggregate, obstruct blood flow, and release mediators that worsen inflammation and cause tissue damage. These mediators include enzymes (PLA2, peroxidases, proteases) and ROS, which likely act together. We observed increased MPO activity and MDA in the gastric tissue of LPS-injected rats, which was partially blocked by PTX. A strong correlation between MPO and MDA levels suggests lipid peroxidation may involve infiltrating leukocytes and their free radicals. Similarly, an increase in lipid peroxidation products, such as MDA, and an increase in tissue MPO activity have been observed in experimental models of intestinal ischemia [33], indicating that at least part of the effect of PLA2 on intestinal ischemic mucosal injury is mediated by products other than arachidonic acid metabolites. Additionally, PLA2 activation, a key enzyme in producing inflammatory agents and toxic lipids, may drive some pro-inflammatory effects of leukocytes. Our results showed an increase in PLA2 activity in gastric mucosa in LPS-challenged rats, which would justify, in part, the decrease in gastric mucosal PC concentration observed in this experimental model.

LPS stimulates the ability of macrophages to produce arachidonic acid metabolites, either through the cyclooxygenase pathway, resulting in the synthesis of various prostaglandins and thromboxanes, or through the lipoxygenase pathway, leading to the production of leukotrienes. This effect may be mediated by the activation of PLA2 due to the action of cytokines or other mediators. In this study, LPS administration did not significantly alter PGE2 levels compared to controls, nor were changes observed with PTX treatment. TXA2 and LTB4 generally promote gastric mucosal damage [34,35]. Our results showed a significant increase in TXB2 levels in the gastric mucosa after LPS exposure, which may contribute to gastric damage through its effects on gastric microcirculation, such as vasoconstriction and platelet aggregation. Additionally, TXB2 induces ROS production by neutrophils, which is linked to stress ulcer pathogenesis. ROS-trapping substances have been shown to reduce stress ulcer formation in sepsis and hemorrhagic shock situations [36]. LTB4 not only stimulates ROS production but also increases neutrophil chemotaxis, adhesion, and vascular permeability. Our study found a significant rise in gastric tissue LTB4 levels following LPS administration, which may contribute to ulcer formation. This effect might involve bile acids, as selective lipooxygenase inhibitors have been shown to protect the gastric mucosa against bile acids [37], supporting the hypothesis that leukotrienes mediate the gastroerosive action of bile acids. In addition, LPS administration significantly reduced PGI2 levels in the gastric mucosa compared to controls, potentially promoting ulcer development by causing vasoconstriction and ischemia, key factors in stress ulcer formation. Similar to coronary artery disease [38], this reduction might result from impaired transfer of endoperoxide precursors between platelets and endothelial cells, leading to increased TXA2 and decreased PGI2 in the endothelial cells.

LPS administration increased plasma NO levels in the LPS + Saline group at 120 min (total of 240 min of procedure), which was reversed by PTX. PTX’s ability to prevent gastric mucosal injury may partly involve altering NO production, though the mechanism is unclear. Excessive NO production during sepsis contributes to vasodilation and hypotension. In the gastric mucosa, physiological NO helps regulate blood flow and maintain mucosal integrity. NOS inhibitors reduced mucosal blood flow increases induced by pentagastrin, indicating NO role in stimulating blood flow. Local NO administration can also reduce ethanol-induced mucosal damage. NO works with prostacyclin and vasodilatory neuropeptides to maintain mucosal integrity. Inhibiting NO synthesis after depleting neuropeptides or blocking prostaglandin synthesis causes mucosal injury [39,40].

LPS injection significantly increased total NOS activity in gastric mucosa, and PTX counteracted this effect. This increase could be attributed to iNOS, as LPS and PTX did not affect cNOS activity. PTX inhibition of iNOS and NO production could be clinically important. Our results also showed that LPS exposure significantly increased CO levels in gastric lavage compared to control groups, an effect partially reversed by PTX. These results may be explained by mucosal infiltration by neutrophils after LPS administration, leading to the generation of ROS and membrane lipid peroxidation. This also may explain the PTX protective mechanism, as it can reduce leukocyte infiltration [41], thereby decreasing ROS generation. Both NO and CO are diffusible gases, and increased levels in gastric lavage may result from elevated plasma levels facilitating their passage into the gastric lumen. However, we believe the increase was due to local production, as plasma levels showed no significant changes. After LPS treatment, we observed a significant rise in tissue cGMP, likely due to increased iNOS activity and NO overproduction, along with elevated lipid peroxidation leading to higher CO levels. PTX reversed these effects. PTX, a phosphodiesterase inhibitor, increases AMP levels without affecting cGMP [42]. Thus, the balance between these nucleotides may be more important in tissue damage than their individual levels.

## 5. Conclusions

To understand the potential protective effect of PTX on the gastric mucosa in our experimental model, several considerations are necessary: (1) Evidence increasingly suggests that PTX significantly impacts cellular mediators of inflammation and tissue damage. It has been shown to reduce inflammation by inhibiting the production of TNF-α, a key inflammatory cytokine, leading to improvements in cytokine-mediated systemic inflammatory states and organ function markers in conditions including nonalcoholic steatohepatitis, coronary artery disease or cholestasis-induced renal injury [43,44,45]. It has also been explored as a treatment for cytokine storms, particularly because of its ability to reduce TNF-α production at the mRNA and bioactivity levels; (2) PTX has shown benefits in experimental models of septic shock, attenuating iNOS expression in combination with albumin [9] or decreasing serum levels of TNF-α and IFN-γ [46]; (3) Protective effect of PTX against cytokines may involve increasing intracellular AMP levels by inhibiting phosphodiesterase, which plays a role in reducing damage to various organs. In experimental gastric ulcers, a significant drop in the cAMP/cGMP ratio, mainly due to increased cGMP, has been observed, alongside a decrease in cAMP levels [47]. Another hypothesis is that PTX induces PGI2 secretion in the vascular endothelium, protecting adjacent cells and contributing to gastric mucosal protection [48]. Although the exact mechanism is unclear, it is believed that PTX vasodilatory effect on gastric microcirculation may be mediated by an increased AMP/cGMP ratio; and (4) PTX is a potent inhibitor of free radical formation, which is involved in mediating some cytokine effects. It prevents acute gastric mucosal injury by reducing neutrophil adhesion and TNF-α levels without affecting mucosal prostaglandin concentrations. Additionally, PTX also inhibits thrombin-stimulated platelet PLA2, which is crucial in the inflammatory process during sepsis and stress ulcer development [49]. As previously mentioned, PLA2 is an important regulator in the formation of different intermediary products, with key functions in the inflammatory process that occurs in states of sepsis, as well as in the cascade of events that occur in stress ulceration.

Despite the positive results of PTX on the sepsis model employed, the following limitations of the study should be mentioned: Firstly, the specific dose and timing of PTX administration may not reflect all clinically relevant regimens. Different doses or schedules might have yielded varying results. Although a broad set of biomarkers has been tested, these may not capture all relevant aspects of PTX mucosal protection or systemic effects. Thus, it would have been of interest to demonstrate the effect on macroscopic gastric mucosa (e.g., number and extension of ulcers) or the improvement of microscopic inflammation (e.g., neutrophil count) after PTX administration, as well as changes in both transcription and translation of the molecules measured in the study. Moreover, functional gastric parameters, such as acid secretion or motility, were not assessed, potentially overlooking key aspects of mucosal health. Finally, the exact mechanisms by which PTX exerts its protective effect should be fully elucidated, necessitating further studies to explore underlying pathways.

Overall, PTX appears to provide substantial protection against acute gastric mucosal injury induced by sepsis in the experimental model used in this study, restoring the production of PC and inhibiting the production of other possible injurious mediators, with various mechanisms involved that require further investigation.

## Data Availability

The data that support the findings of this study are available from the corresponding author upon reasonable request.
